# FXTAS is rare among Portuguese patients with movement disorders: *FMR1 *premutations may be associated with a wider spectrum of phenotypes

**DOI:** 10.1186/1744-9081-7-19

**Published:** 2011-06-03

**Authors:** Ana I Seixas, José Vale, Paula Jorge, Isabel Marques, Rosário Santos, Isabel Alonso, Ana M Fortuna, Jorge Pinto-Basto, Paula Coutinho, Russell L Margolis, Jorge Sequeiros, Isabel Silveira

**Affiliations:** 1 UnIGENe, IBMC - Instituto de Biologia Molecular e Celular, Universidade do Porto, Porto, Portugal; 2 Department of Neurology, Hospital de Egas Moniz, Lisboa, Portugal; 3 Centro de Genética Médica Dr. Jacinto Magalhães, INSA, Porto, Portugal; 4 Department of Neurology, Hospital de São Sebastião, Feira, Portugal; 5 Division of Neurobiology, Department of Psychiatry, Johns Hopkins University School of Medicine, Baltimore, USA; 6 ICBAS, Universidade do Porto, Portugal

## Abstract

The fragile X-associated tremor/ataxia syndrome (FXTAS) is a late-onset neurodegenerative disorder caused by expansions of 55-200 CGG repeats in the 5'UTR of the *FMR1 *gene. These *FMR1 *premutation expansions have relatively high frequency in the general population. To estimate the frequency of *FMR1 *premutations among Portuguese males with non-familial, late-onset movement disorders of unknown etiology, we assessed CGG repeat size in males with disease onset after the age of 50 and negative or unknown family history for late-onset movement disorders, who were sent for SCA, HD, or PD genetic testing at a reference laboratory. The selected patients had a primary clinical diagnosis based on one of the following cardinal features of FXTAS: ataxia, tremor, or cognitive decline. A total of 86 subjects were genotyped for the CGG repeat in the *FMR1 *gene. We detected one patient with an expansion in the premutation range. The frequency of *FMR1 *premutations was 1.9% (1/54) in our group of patients with ataxia as the primary clinical feature, and 1.2% (1/86) in the larger movement disorders group. In the family of the FXTAS case, premutation-transmitting females presented a history of psychiatric symptoms, suggesting that, given the wide phenotypical expression of the premutation in females, neuropsychiatric surveillance is necessary. In conclusion, genetic testing for FXTAS should be made available to patients with adult-onset movement disorders to enable adequate genetic counseling to family members.

## Findings

Expansions of a CGG repeat tract in the 5'UTR region of the *FMR1 *gene causes the fragile X syndrome (FXS), the most common inherited cause of mental retardation. FXS is an X-linked disorder, characterized by moderate to severe mental impairment, facial dysmorphism and behavioral abnormalities in males, and by milder symptoms in some carrier females [1]. Full mutations (more than 200 CGGs) cause FXS, as they lead to loss of function due to gene silencing mediated by abnormal methylation patterns [2,3]. Normal alleles range in length from 5 to 44 triplets, and repeats of 45-54 CGGs are "grey zone alleles" of unknown biological significance [4]. Premutations range from 55 to 200 CGGs and have been associated with two different phenotypes: females are at a higher risk for premature ovarian insufficiency (POI) [5]; whereas males may be affected by the fragile X-associated tremor/ataxia syndrome (FXTAS) [6,7].

FXTAS is characterized by progressive cerebellar ataxia, tremor, and parkinsonism with bradikynesia and rigidity; other symptoms may include cognitive decline and peripheral neuropathy [8]. Neuroradiological findings show generalized brain atrophy with white matter lesions in the middle cerebellar peduncle [9,10]. Clinical criteria for FXTAS were proposed by Hagerman et al. [11]: presence of a *FMR1 *premutation, one major clinical sign (gait ataxia or intention tremor), and one major finding on neuroimaging (white matter lesions in middle cerebellar peduncles). Histopathology of FXTAS postmortem brains revealed loss of Purkinje cells in the cerebellum, and presence of eosinophilic intranuclear inclusions in neurons and astrocytes of the cortex [12]. The pathogenic mechanism of FXTAS seems to be different from that of FXS: abnormal methylation does not occur with premutation and, therefore, a loss of function mechanism is unlikely. Instead, it appears to be associated with toxic RNA species containing long CGG tracts [13].

Penetrance of FXTAS in males carrying *FMR1 *premutations increases from 17% in the sixth decade to 75% after the age of 80. FXTAS in females has been reported [14,15], but appears to be rare. Though only 1/4000 men have the full mutation, frequency of premutations is much greater: 1/1000 in Caucasian males [16]. The high frequency of *FMR1 *premutations in the general population was taken as an indication that they could represent a major cause of non-familial cases of late-onset ataxia. A review of the literature reported that the frequency of *FMR1 *premutations, in all combined ataxia cases, was 1.3% in men and 0.24% in women [17]. As ataxia in FXTAS is often associated with tremor and/or cognitive decline, it is plausible that some cases of FXTAS could present for testing with a clinical diagnosis other than cerebellar ataxia; therefore, other movement disorders have also been screened for *FMR1 *premutations. The combination of all studies published up to 2008, in patients with movement disorders other than ataxia (including essential tremor, parkinsonism, and multiple system atrophy), gives a total frequency of CGG repeat length in the FXTAS range of 0.37%, higher than the overall population rate [17].

Here, we present the first study on the frequency of *FMR1 *premutations in a sample of Portuguese males sent for genetic testing, with late-onset movement disorders characterized by one or more of the FXTAS cardinal clinical features (ataxia, tremor, or cognitive decline). Additionally, we investigated transmission of the *FMR1 *repeat among FXTAS family relatives.

Cases were ascertained from a pool of patients referred to a reference laboratory in Portugal for genetic testing for neurological diseases. Inclusion criteria were as follows: males with onset after age 50 years, absent or unknown family history of autosomal dominant late-onset movement disorders, clinical diagnosis of late-onset movement disorder (spinocerebellar ataxia, SCA; Huntington disease, HD; or Parkinson disease, PD), and absence of SCA, HD, or relevant PD mutations. Only those presenting with ataxia, parkinsonism, and/or cognitive decline were included. Genomic DNA was isolated from peripheral blood using standard techniques. All patients gave their informed consent. Assessment of (CGG)_n _in *FMR1 *was performed by PCR, improved for amplification of GC-rich sequence regions, as described previously [18]. PCR products were visualized on agarose gel stained with ethidium bromide and compared to a control with 30 CGGs; PCR products that appeared larger were further analyzed by automated fragment analysis in a 3730*xl *DNA Analyzer (Applied Biosystems, Foster City, CA) for assessment of repeat number. Premutation status was established for alleles with 55-200 CGGs. Full mutations were detected by double digestion with *EcoR*I and *Eag*I of 10 μg of genomic DNA, followed by electrophoresis in agarose gels and blotting onto Hybond N+ membranes (Amersham, UK). Probe Ox1.9 was labeled by P^32^-dCTP nick translation and hybridized according to standard methodology. Autoradiography was carried out for 24-48 hours at -70°C, using intensifying screens and Kodak X-Omat-RP films.

A total of 86 male patients were identified as candidates, following application of the defined criteria. Demographics and clinical features are summarized in Table 1; 54/86 patients (63%) had ataxia, and 50/86 (58%) had no known family history of late-onset movement disorders. We identified one patient with approximately 95 CGGs. Frequency of premutations in the total sample was 1.2%. In the subset of patients with ataxia, frequency was 1.9%.

**Table 1:** Demographics and clinical features of the patient population

	Cardinal clinical feature			
		
	Ataxia (n = 54)	Tremor (n = 4)	Cognitive deficits (n = 28)	*Total* (N = 86)
Age (mean ± SD)	67.7 ± 9.1	59.0 ± 8.6	70.0 ± 9.2	68.0 ± 9.3
Age-of-onset (mean ± SD)	59.0 ± 6.8	54.0 ± 1.4	60.5 ± 6.7	59.2 ± 6.7
With additional cardinal features (*n*)	13	1	4	18
Family history (*n*)				
*None*	33	3	14	50
*Unknown*	21	1	14	36

**Figure 1:**
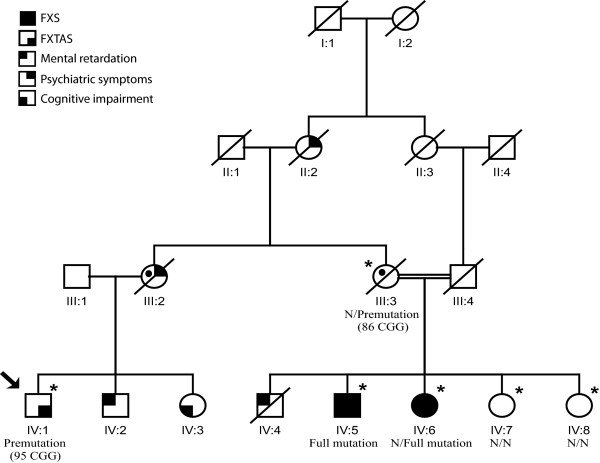
**Family pedigree of the FXTAS patient**. There is family history of mental retardation in males, and psychiatric disease in females. Individual IV:3 also presents tremor. Individual IV:4 died in infancy and was never tested for FXS. *FMR1 *repeat lengths are available for individuals marked with an asterisk. Symbols with the black circle are obligatory carriers of a *FMR1 *expanded allele. An arrow indicates the proband. (N, normal repeat size).

The patient carrying the *FMR1 *premutated allele was a 73-year-old male, who reported onset at age 51, and was sent for genetic testing of SCAs (Figure 1). He had an initial clinical diagnosis of essential tremor that did not improve upon treatment with beta-blockers and antiepileptic drugs. Further examination at age 66 revealed a mild cerebellar syndrome with gait ataxia and dysarthria, difficulties with memory and executive function, and neurosensorial deafness. MRI showed diffuse atrophy of the cerebellum and cerebellar peduncles. At age 72, the patient presented with aggravated cognitive deficits. These clinical features and radiological findings confirmed a probable FXTAS diagnosis.

After identification of this FXTAS patient, we studied the family and discovered a clinical history of FXS (Figure 1) in two first cousins of the proband (IV:5 and IV:6). Information provided by the proband's son and by sisters of the FXS patients also suggests a history of psychiatric disease among premutation-transmitting females; III:2 had an admission to a psychiatric hospital. In addition, IV:3 was reported to have a tremor and cognitive deficits, but no neurological exam or DNA were available. Consanguinity was present in the family, thus increasing the risk for genetic diseases. No additional information was obtained for this family. Two patients carrying 'grey zone' alleles of 49 and 52 repeats (a frequency of 2.3% in the total sample) were also found. In the general population, the frequency of grey zone alleles is 3-4/100 males [16,19].

This study presents the first FXTAS screening in the Portuguese population of males with adult-onset movement disorders sent for genetic testing. The frequency of *FMR1 *premutations was 1.9% (1/54) in patients with ataxia as the cardinal clinical feature, which is within the range reported by multiple studies (0% to 4.1%), and 1.2% (1/86) in our global sample. A review of all such studies (Table 2) shows that the *FMR1 *premutation rate among men with cerebellar syndromes averages 1.2%. Ascertainment of subjects, criteria used and the population studied may explain some of the frequency variability. As ascertainment via test request for SCA could fail to detect other FXTAS cases, we also included individuals referred for testing for PD and HD. We also applied stringent criteria, as only males with onset above age 50 years and no suggestive family history of late-onset movement disorders were included. However, we found no *FMR1 *premutations in 32 patients with a movement disorder other than ataxia, which is consistent with the literature (Table 2). Only 3 premutation carriers (a rate of 0.3%) have been found in movement disorder cohorts that exclude ataxia cases - two had an initial diagnosis of multisystem atrophy (important in the differential diagnosis of ataxia), while the other was referred for HD testing.

**Table 2:** Frequency of *FMR1 *premutations among male patients with adult-onset movement disorders

Sample origin	Ascertainment via	Inclusion criteria	Premutation rate	Study
United Kingdom	Referral for genetic test of SCA	Ataxia	2/59	(Macpherson et al., 2003) [23]
USA	Clinical diagnosis of ET	ET	0/40	(Garcia Arocena et al., 2004) [24]
USA	Clinical diagnosis of MSA	MSA	0/40	(Garland et al., 2004) [25]
USA	Referral for genetic test of SCA	Ataxia; age > 50	1/167	(Milunsky and Maher 2004) [26]
Singapore	Clinical diagnosis of movement disorder	ET; age > 45	0/34	(Tan et al., 2004) [27]
		Ataxia; isolated	0/30	
		MSA	0/12	
		APD	0/15	
Japan	Clinical diagnosis of MSA	MSA	0/36	(Yabe et al., 2004) [28]
Germany	Referral for genetic test of SCA	Ataxia; AOO > 50	0/269	(Zuhlke et al., 2004) [29]
Europe (mixed)	Clinical diagnosis of MSA or related	MSA	0/76	(Biancalana et al., 2005) [30]
		Ataxia	1/19	
Italy	Clinical diagnosis of SCA	Ataxia	6/275	(Brussino et al., 2005) [31]
Europe (mixed)	Clinical diagnosis of MSA	MSA*	2/253	(Kamm et al., 2005) [32]
USA	Referral for genetic test of SCA and HD	Cerebellar disease	1/73	(Seixas et al., 2005) [18]
		Basal ganglia disease	0/6	
Mixed	Clinical diagnosis of PD	PD	0/414	(Toft et al., 2005) [33]
Belgium	Referral for genetic test of SCA	Ataxia; age > 50	5/122	(Van Esch et al., 2005) [34]
Spain	Clinical diagnosis of SCA	Ataxia; isolated; age > 45	1/87	(Rodriguez-Revenga et al., 2007) [35]
USA	Referral for genetic test of SCA	Ataxia; age > 50	1/286	(Adams et al., 2008) [17]
Poland	Clinical diagnosis of SCA	Ataxia; age > 50	1/178	(Rajkiewicz et al., 2008) [36]
Brazil	Clinical diagnosis of movement disorder	Ataxia, and/or tremor, and/or parkinsonism; age > 45	0/66	(Reis et al., 2008) [37]
Spain	Referral for genetic test of HD	HD	1/95	(Rodriguez-Revenga et al., 2008) [38]
United Kingdom	Clinical diagnosis of SCA	Ataxia	0/105	(Wardle et al., 2009) [39]
Portugal	Referral for genetic test of SCA, HD, and PD	Ataxia; isolated; AOO > 50	1/54	This study
		Tremor or cognitive decline	0/32	
				

*Total of ataxia referrals*			20/1724	
*Total of movement disorder referrals (other than ataxia)*			3/1119	

SCA, spinocerebellar ataxia; ET, essential tremor; MSA, multisystem atrophy; PD, Parkinson disease; APD, atypical Parkinson disease; AOO, age of onset. *Includes proven, possible, and probable MSA

Our study provides further evidence that a primary diagnosis of cerebellar ataxia is still the cardinal feature of the vast majority of FXTAS cases. There has been debate as to whether genetic testing for FXTAS should be included in screening panels for late-onset movement disorders. We conclude that, though the prevalence of FXTAS appears low, genetic testing should be made available to patients with late-onset movement disorders, particularly with a clinical diagnosis of ataxia, and in agreement with the guidelines suggested by Berry-Kravis et al. [9].

FXTAS appears to be quite rare among women carrying premutations, but a recent study describes a higher rate of neuropsychiatric symptoms among daughters of FXTAS patients, including memory and balance problems, tremor, and psychiatric disturbances [20]. Motor and mental dysfunction was recently reported in a FXTAS premutation carrier in a mother-daughter transmission [21]. Another study identified a high lifetime risk of mood and anxiety disorders among male and female carriers of the *FMR1 *premutation [22]. These findings concur with our observation that, in the family of the Portuguese FXTAS patient, there is an increased risk for motor and/or mental dysfunction in relatives carrying the premutation.

In light of this, and given the complex phenotypic expression of the *FMR1 *expansions, psychological and neurologic surveillance (in addition to screening for POI in females) may be necessary for relatives of FXS and FXTAS patients, and perhaps for carriers in general. The complex inheritance and wide spectrum of phenotypes associated with the *FMR1 *(CGG)_n _reinforces the importance of genetic counseling in these families.

## Competing interests

The author declares that they have no competing interests.

## Authors' contributions

Study concept and design: AIS and IS. Acquisition of data: AIS, JV, IM, RS, IA, AMF, JPB and PC. Critical revision of the manuscript for important intellectual content: AIS, RLM, JS and IS. Study supervision: IS. All authors read and approved the final manuscript.
